# Renal cell carcinoma incidence and mortality in California: a population-based study of sociodemographic patterns and temporal trends from 1988 through 2019

**DOI:** 10.1016/j.lana.2026.101430

**Published:** 2026-02-27

**Authors:** Kevin L'Espérance, Katherine Lin, Daphne Lichtensztajn, Simon John Christoph Soerensen, Shuchi Gulati, Zhengyi Deng, John T. Leppert, David Y. Oh, Lori C. Sakoda, Samuel L. Washington, Maxwell V. Meng, June M. Chan, Marvin E. Langston, Iona Cheng, Benjamin I. Chung, Rebecca E. Graff

**Affiliations:** a Department of Urology, Stanford University School of Medicine, Palo Alto, CA, USA; b Department of Epidemiology and Population Health, Stanford University School of Medicine, Palo Alto, CA, USA; c Department of Epidemiology and Biostatistics, University of California, San Francisco, San Francisco, CA, USA; d Division of Hematology and Oncology, Department of Internal Medicine, University of California Davis, Sacramento, CA, USA; e Division of Nephrology, Department of Medicine, Stanford University School of Medicine, Palo Alto, CA, USA; f Division of Urology, Veterans Affairs Palo Alto Health Care System, Palo Alto, CA, USA; g Division of Hematology/Oncology, Department of Medicine, University of California, San Francisco, San Francisco, CA, USA; h Division of Research, Kaiser Permanente Northern California, Pleasanton, CA, USA; i Department of Health Systems Science, Kaiser Permanente Bernard J. Tyson School of Medicine, Pasadena, CA, USA; j Department of Urology, University of California, San Francisco, San Francisco, CA, USA; k Helen Diller Family Comprehensive Cancer Center, University of California, San Francisco, CA, USA

**Keywords:** Kidney cancer, Renal cell carcinoma, Incidence, Mortality, Trends, California

## Abstract

**Background:**

Renal cell carcinoma (RCC) incidence and mortality trends in California that reflect contemporary patterns of incidental diagnoses and new treatment advances have not been published, and RCC burden across sociodemographic groups remains unclear. We sought to investigate the incidence and mortality rates of first RCC in individuals aged 20 and older in California from 1988 through 2019, including overall trends and by sociodemographic factors.

**Methods:**

Data were obtained from the California Cancer Registry. We calculated age-adjusted incidence and mortality rates and rate ratios (where relevant) of RCC overall and by sex, age, race and ethnicity, and neighborhood socioeconomic status (nSES). Joinpoint regression was used to estimate annual percent rate changes in incidence and mortality rates across time periods.

**Findings:**

From 1988 through 2019, we identified 90,659 incident RCC cases, of which 62.9% were localized at diagnosis, and 37,069 RCC-related deaths. Incidence was higher in males than females, among American Indian/Alaska Native, Hispanic, and non-Hispanic Black individuals, and in lower-nSES areas. Incidence rose over time, with steeper increases among Hispanic and non-Hispanic Black populations and in the lowest nSES groups. Although mortality declined overall, disparities persisted, with Hispanic and non-Hispanic Black individuals experiencing higher mortality than non-Hispanic White individuals.

**Interpretation:**

RCC remains a significant public health concern in California, with disparities by race, ethnicity, and nSES widening over time. This underscores the relevance of RCC as a health equity issue and the need for targeted public health action and equity-focused research.

**Funding:**

This study utilized publicly accessible data from infrastructure supported by the California Department of Public Health, the National Cancer Institute, and the Centers for Disease Control and Prevention.

**Research in context**

**Evidence before this study**

Renal cell carcinoma (RCC) is an important but understudied health concern in the United States. With its large, racially and socioeconomically diverse population and long-standing, high-quality cancer registry, California offers a unique setting for examining temporal trends in RCC incidence and mortality. To assess existing evidence, MEDLINE was searched through May 1, 2025, using the following terms: (“kidney cancer” OR “renal cell carcinoma”) AND (“incidence” OR “mortality”) AND (“trends”) AND (“California”). In California, one prior analysis of RCC described trends in incidence and mortality. However, this study predated the widespread adoption of targeted therapies and immune checkpoint inhibitors, which have substantially improved survival. Additionally, the previous work did not assess differences in disease burden across sociodemographic groups. Consequently, contemporary data were needed to capture current RCC trends and inequities in burden across the diverse California population.

**Added value of this study**

We leveraged data from the California Cancer Registry to update and assess RCC incidence and mortality from 1988 to 2019. We examined trends by sociodemographic factors to identify inequities and provide insights into population groups that could benefit from improved prevention, detection, and treatment strategies. Overall, we observed widening disparities in RCC incidence and mortality over time, particularly between low- and high-SES neighborhoods. We also observed increasing disparities in incidence among Non-Hispanic Black and Hispanic individuals compared to Non-Hispanic White individuals.

**Implications of all the available evidence**

Widening disparities in RCC incidence and mortality in California highlight the urgent need for targeted interventions that address social, structural, and environmental drivers of risk. Strengthening surveillance and integrating multi-level data will be critical to guide policies and ensure equitable improvements in prevention, detection, and survival.

## Introduction

Kidney cancer is an important but understudied health concern in the United States (US), with an estimated 80,980 new cases and 14,510 deaths in 2025.^1^ US data from 2010 through 2023 indicate that age-adjusted kidney cancer incidence rates peaked around 2019 and have since stabilized, mirroring trends observed in other high-income countries.^2^^,^^3^ Mortality rates have shown a promising downward trend, with an average annual decline of 0.6% between 1999 and 2020.^4^ This encouraging change likely signals progress in smoking reduction, advancements in medical treatments (e.g., introduction of immune checkpoint inhibitors and targeted therapy), and other improvements in patient care.^5^^,^^6^

In California, the most populous state in the US, over 7500 new cases of and 1350 deaths from kidney cancer occur each year, with approximately 90% of the cases classified as renal cell carcinoma (RCC).^2^ In a previous California analysis of RCC incidence and mortality trends, the authors reported a sharp rise in RCC incidence through 2009 that stabilized thereafter, while mortality tended to decline throughout.^7^ These conclusions were consistent across groups defined by age, sex, race and ethnicity, and neighborhood socioeconomic status (nSES),^7^ though the authors did not examine differences in the burden of RCC among groups defined by these factors. Since that analysis, the introduction of targeted therapies and immune checkpoint inhibitors has revolutionized RCC treatment, substantially improving survival for many patients.^8^ Thus, there is a need to update these trends with recent data that reflect contemporary patterns of RCC and new treatment advances and to investigate the burden of RCC across sociodemographic groups within California's diverse population.

In this study, we used population-based data from the California Cancer Registry (CCR) to examine the latest trends in RCC incidence and mortality, overall and by age, sex, race and ethnicity, and nSES. Furthermore, we compared the burden of RCC across groups defined by these factors.

## Methods

### Study design and data source

The CCR is a mandatory state-wide cancer surveillance program that has collected information on all cancer cases in California since 1988, including tumor characteristics, demographics, and geocoded addresses at the time of diagnosis. We accessed the CCR to obtain information on all pathologically confirmed primary RCC cases in persons aged 20 and older from January 1, 1988, through December 31, 2019.^9^ We limited the analysis to data collected through 2019, as including cancer incidence data from the COVID-19 pandemic period could have reduced model fit and accuracy of trend estimates, complicating their interpretation.^10^ We identified all cases of RCC using site code C64.9 from the International Classification of Diseases for Oncology (ICD-O-2 until 2000 and ICD-O-3 from 2001 onward) and the following histologic codes: clear cell (8310/3, 8323/3); papillary and hereditary leiomyomatosis-associated (HLRCC, 8260/3, 8311/3); chromophobe (8317/3); sarcomatoid (8318/3); cyst-associated (8316/3); collecting duct (8319/3); medullary (8510/3); and unclassified/not otherwise specified (8312/3). We applied the Surveillance, Epidemiology, and End Results (SEER) Program Staging Rule to determine the diagnostic stage of tumors: localized (confined to the kidney), regional (extending to nearby tissues or lymph nodes), distant (metastasized to distant sites), or unknown (if stage information was unavailable). Initial course of treatment was classified into: partial nephrectomy, total or radical nephrectomy, unspecified type of surgery, no surgery, or unknown. Deaths attributable to kidney cancer, hereafter referred to as RCC (for consistency with the analyses of RCC incidence), through December 31, 2019 were sourced from the California Department of Public Health.^11^ While the mortality data were not specific to RCC, RCC comprises about 90% of kidney cancer cases.^2^ This study used publicly available, de-identified data and was determined not to require institutional review board approval or informed consent at the University of California, San Francisco.

### Procedures

We categorized age at diagnosis into five groups (20–44, 45–54, 55–64, 65–74, 75+ years) and sex as a binary construct (“female” and “male”). Race and ethnicity were categorized into mutually exclusive categories, as follows: “American Indian/Alaska Native,” “Asian/Pacific Islander,” “Hispanic,” “Non-Hispanic Black,” and “Non-Hispanic White.” Cases with other/unknown race/ethnicity (n = 474) were excluded from rate calculations due to the lack of a population denominator. As a proxy of socioeconomic status, geocoded addresses at diagnosis were appended to an established composite nSES index based on principal component analysis of US Census data that incorporated census block group data on household income, education, and occupation, unemployment, poverty, rent, and house values.^12^ Individuals were categorized into quintiles of nSES scores based on the statewide distribution, with quintile 1 representing the group with the lowest nSES.^12^

### Statistical analyses

We used mid-year population counts sourced from the US Department of Finance for 1988–1989 and from the SEER program for 1990–2019 as proxies for person-time at risk, assuming each individual contributed approximately one person-year (p-y) per year (https://seer.cancer.gov/popdata/). We calculated age-adjusted incidence and mortality rates using SEER∗Stat software (version 8.4.5; National Cancer Institute, Bethesda, MD, USA), with the number of incident cases or deaths as the numerator and p-y at risk as the denominator. Rates were standardized to the 2000 US standard population using the direct method, as adopted by federal, state, and local agencies to ensure comparability and consistency.^13^ Incidence rates (IRs) were stratified by tumor-related (i.e., histology and stage) and sociodemographic (i.e., sex, age, race and ethnicity, and nSES) factors. Mortality rates (MRs) were stratified by sex, age, and race and ethnicity; the mortality dataset did not include nSES information. In addition, incidence rate ratios (IRRs) and mortality rate ratios (MRRs) were estimated across categories defined by age (reference: 20–44 years), sex (reference: male), and race and ethnicity (reference: non-Hispanic White), with calculations across nSES categories (reference: low nSES-first quintile) for incidence only. As the nSES database provided estimates starting from 2006, the analysis period for nSES covered 2006 through 2019. 95% confidence intervals were calculated using the Tiwari method.^14^ All IRs and MRs were reported per 100,000 p-y.

We performed analyses of temporal trends with the Joinpoint Regression Program (version 5.3.0, National Cancer institute, Bethesda, MD, US). Joinpoint linear regression describes temporal trends by identifying points of change (“joinpoints”) where the slope of a regression line shifts significantly over time. For each segment, trends in incidence and mortality rates were quantified using estimated annual percent change (APC). The 95% CIs for APC were estimated using the default empirical quantile method.^15^ In addition to overall trend analysis, we analyzed trends in subgroups defined by age, sex, race and ethnicity, and nSES (incidence only). Because joinpoint models select the best fit for each dataset, subgroup analyses could yield different joinpoints from the overall analysis. Due to small sample sizes, trend analyses were not conducted for American Indian and Alaska Native individuals. To provide an overall summary measure across the entire period (1988–2019), we estimated the average annual percent change (AAPC), which represents a weighted average of the APCs from each segment, weighted by the length of each segment.^16^

### Role of the funding source

The funding sources had no role in the study design; data collection, analysis, or interpretation; manuscript preparation; or the decision to submit the manuscript for publication.

## Results

### Tumor-related characteristics

We identified 90,659 incident RCC cases (90.8% of all kidney cancers) statewide from 1988 through 2019 among adults 20 years and older. The mean (SD) age at diagnosis was 62.2 years (13.2). The overall IR in this population was 11.88 (95% CI: 11.80, 11.96) cases per 100,000 p-y. Clear cell was the most common histological subtype, accounting for 79.2% of cases with known histology at an IR of 5.69 (95% CI: 5.63, 5.74) cases per 100,000 p-y (Table 1). This was followed by papillary and HLRCC (7.0%; IR: 0.82; 95% CI: 0.80, 0.82 cases per 100,000 p-y) and chromophobe (4.1%; IR: 0.48; 95% CI: 0.47, 0.50 cases per 100,000 p-y), although many cases were of unspecified histology (39.1%), with an incidence of 4.71 (95% CI: 4.66, 4.75) cases per 100,000 p-y. Approximately two-thirds of cases were localized at diagnosis, with an IR of 7.45 (95% CI: 7.39, 7.52) per 100,000 p-y. In contrast, roughly one-fifth presented with metastasis, at an IR of 2.06 (95% CI: 2.03, 2.10) cases per 100,000 p-y. As primary course of treatment, 54.5% of all cases underwent total or radical nephrectomy, while 23.3% received partial nephrectomy.

**Table 1:** Distribution of tumor-related characteristics and age-adjusted incidence rates of primary renal cell carcinoma, California Cancer Registry, 1988 to 2019.

Characteristics	*n* cases (%)	IR (95% CI)^a^
All	90,659 (100.0)	11.9 (11.80–11.96)
Histology		
Clear cell	43,710 (48.2)	5.69 (5.63, 5.74)
Papillary & HLRCC	6337 (7.0)	0.82 (0.80, 0.82)
Chromophobe	3702 (4.1)	0.48 (0.47, 0.50)
Sarcomatoid	952 (1.1)	0.12 (0.12, 0.13)
Cyst-associated	297 (0.3)	0.04 (0.03, 0.04)
Collecting duct	140 (0.2)	0.02 (0.02, 0.02)
Medullary	30 (<0.1)	0.00 (0.00, 0.00)
Unspecified	35,491 (39.1)	4.71 (4.66, 4.75)
Stage		
Localized	57,059 (62.9)	7.45 (7.39, 7.52)
Regional	14,580 (16.1)	1.91 (1.88, 1.94)
Distant	15,703 (17.3)	2.06 (2.03, 2.10)
Unknown	3317 (3.7)	0.45 (0.44, 0.47)
Primary treatment		
Partial Nephrectomy	21,101 (23.3)	
Total/Radical Nephrectomy	49,399 (54.5)	
Surgery not otherwise specified	2186 (2.4)	
No surgery	17,388 (19.2)	
Unknown	585 (0.6)	

Abbreviations: CI, confidence interval; HLRCC, hereditary leiomyomatosis-associated renal cell carcinoma; IR, incidence rate; RCC, renal cell carcinoma.

a Incidence rates per 100,000 person-years age-standardized to the 2000 United States population.

### Sociodemographic factors and RCC incidence and mortality

Incidence of RCC was higher in males than females, with females having a 51% lower IR (IRR: 0.49; 95% CI: 0.48, 0.50) (Table 2 and Supplementary Figure S1). Incidence largely increased with age, peaking in the 65–74 age group compared to those aged 20–44 (IRR: 14.67; 95% CI: 14.31, 15.03). In comparison to non-Hispanic White individuals, the IRRs were 0.62 (95% CI: 0.61, 0.64) for Asian or Pacific Islander, 1.46 (95% CI: 1.35, 1.57) for American Indian or Alaska Native, 1.32 (95% CI: 1.30, 1.34) for Hispanic, and 1.21 (95% CI: 1.18, 1.24) for non-Hispanic Black individuals. During the period from 2006 to 2019 (55,916 RCC cases), RCC incidence decreased as levels of nSES increased (*p*-trend: 0.008), with the highest quintile showing a 28% lower IR compared to the lowest quintile (IRR: 0.72; 95% CI: 0.70, 0.74). This gradient across levels of nSES was observed for all RCC stages (Supplementary Table S1).

**Table 2:** Age-adjusted primary renal cell carcinoma incidence rates and incidence rate ratios for sociodemographic factors, California Cancer Registry, 1988–2019.

Sociodemographic variables	*n* cases (%)	*n* population (%)	IR (95% CI)^a^	IRR (95% CI)
Sex				
Male	58,048 (64.1)	393,239,162 (49.3)	16.37 (16.24, 16.51)	1.00 (ref.)
Female	32,611 (35.9)	404,449,618 (50.7)	8.02 (7.93, 8.11)	0.49 (0.48, 0.50)
Age at diagnosis (years)				
20–44	8759 (9.7)	423,917,497 (53.1)	2.28 (2.23, 2.33)	1.00 (ref.)
45–54	16,489 (18.2)	140,753,774 (17.6)	11.68 (11.50, 11.86)	5.13 (5.00, 5.27)
55–64	24,836 (27.3)	105,087,095 (13.3)	23.57 (23.28, 23.87)	10.35 (10.10, 10.61)
65–74	23,648 (26.1)	70,932,310 (8.9)	33.39 (32.97, 33.82)	14.67 (14.31, 15.03)
75+	16,927 (18.7)	56,998,104 (7.1)	29.94 (29.49, 30.40)	13.15 (12.81, 13.50)
Race and ethnicity				
Non-Hispanic White	52,547 (58.3)	401,195,991 (50.2)	11.50 (11.40, 11.60)	1.00 (ref.)
American Indian/Alaska Native	734 (0.8)	4,712,321 (0.6)	16.76 (15.53, 18.07)	1.46 (1.35, 1.57)
Asian or Pacific Islander	6993 (7.8)	105,921,864 (13.3)	7.16 (7.00, 7.34)	0.62 (0.61, 0.64)
Hispanic	23,587 (26.1)	235,045,052 (29.5)	15.15 (14.95, 15.36)	1.32 (1.30, 1.34)
Non-Hispanic Black	6324 (7.0)	50,813,552 (6.4)	13.91 (13.56, 14.26)	1.21 (1.18, 1.24)
Quintiles of nSES^b^				
1 (lowest)	9834 (17.7)	69,378,626 (17.9)	16.18 (15.86, 16.51)	1.00 (ref.)
2	11,268 (20.2)	75,703,841 (19.6)	15.21 (14.93, 15.50)	0.94 (0.91, 0.97)
3	12.175 (21.9)	81,015,508 (20.9)	14.47 (14.22, 14.74)	0.89 (0.87, 0.92)
4	11,887 (21.3)	81,687,486 (21.1)	13.62 (13.37, 13.87)	0.84 (0.82, 0.86)
5 (highest)	10,547 (18.9)	78,890,086 (20.4)	11.65 (11.43, 11.88)	0.72 (0.70, 0.74)

Abbreviations: CI, confidence interval; IR, incidence rate; IRR, incidence rate ratio; nSES, neighborhood socioeconomic status.

a Incidence rates per 100,000 person-years age-standardized to the 2000 United States population.

b Because the coverage period was from 2006 to 2019, the numbers do not add up to the total for the period from 1988 to 2019.

A total of 37,069 deaths from RCC were recorded from 1988 through 2019. The mean (SD) age at kidney cancer death was 69.8 (13.2). RCC mortality was 56% lower in females compared to males (MRR: 0.44; 95% CI: 0.43, 0.45) (Table 3 and Supplementary Figure S2). Mortality increased with age, with older individuals having a higher risk of RCC death compared to those within the 20–44 age group. Relative to non-Hispanic White individuals, mortality was higher among American Indian or Alaska Native (MRR: 1.80; 95% CI: 1.60, 2.01), Hispanic (MRR: 1.11; 95% CI: 1.08, 1.14), and non-Hispanic Black (MRR: 1.06; 95% CI: 1.01, 1.11) individuals, but lower among Asian or Pacific Islander individuals (MRR: 0.54; 95% CI: 0.52, 0.57).

**Table 3:** Age-adjusted primary renal cell carcinoma mortality rates and mortality rate ratios for sociodemographic factors, California Cancer Registry, 1988–2019.

Sociodemographic variables	*n* deaths (%)	*n* population	MR (95% CI)^a^	MRR (95% CI)
Sex				
Male	23,766 (64.1)	393,239,162 (49.3)	7.29 (7.20, 7.39)	1.00 (ref.)
Female	13,303 (35.9)	404,449,618 (50.7)	3.20 (3.15, 3.26)	0.44 (0.43, 0.45)
Age at diagnosis (years)				
20–44	1199 (3.2)	423,917,497 (53.2)	0.31 (0.30, 0.33)	1.00 (ref.)
45–54	3580 (9.7)	140,753,774 (17.6)	2.53 (2.45, 2.62)	8.07 (7.56, 8.63)
55–64	7690 (20.7)	105,087,095 (13.3)	7.29 (7.12, 7.45)	23.22 (21.84, 24.70)
65–74	10,088 (27.2)	70,932,310 (8.9)	14.39 (14.11, 14.67)	45.86 (43.18, 48.74)
75+	14,512 (39.2)	56,998,104 (7.1)	25.23 (24.82, 25.64)	80.40 (75.78, 85.37)
Race and ethnicity				
Non-Hispanic White	24,416 (65.9)	401,195,991 (50.2)	5.15 (5.09, 5.22)	1.00 (ref.)
American Indian/Alaska Native	340 (0.9)	4,712,321 (0.6)	9.25 (8.25, 10.34)	1.80 (1.60, 2.01)
Asian or Pacific Islander	2526 (6.8)	105,921,864 (13.3)	2.80 (2.69, 2.91)	0.54 (0.52, 0.57)
Hispanic	7473 (20.2)	235,045,052 (29.5)	5.72 (5.58, 5.85)	1.11 (1.08, 1.14)
Non-Hispanic Black	2283 (6.2)	50,813,552 (6.4)	5.46 (5.23, 5.69)	1.06 (1.01, 1.11)

Abbreviations: CI, confidence interval; MR, mortality rate; MRR, mortality rate ratio.

a Mortality rates per 100,000 person-years age-standardized to the 2000 United States population.

### Temporal trends in RCC incidence and mortality

**Fig. 1:**
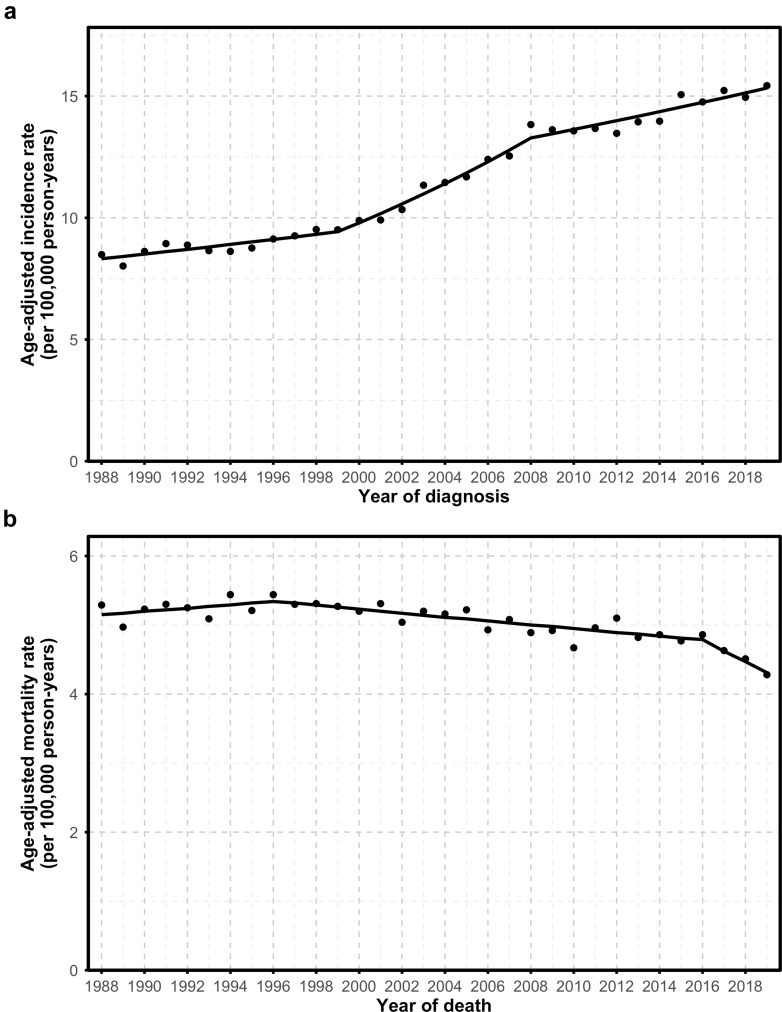
**Trends in renal cell carcinoma incidence (a) and mortality (b) rates, California Cancer Registry, 1988–2019**.

APCs (95% CIs) for trends are provided in Table 4. From 1988 through 2019, RCC incidence trends showed three distinct phases, with an increase from 1988 to 1999 (APC: 1.15%; 95% CI: −0.17, 1.82), a sharper rise from 1999 to 2008 (APC: 3.88%; 95% CI: 3.19, 6.71), and another increase from 2008 to 2019 (APC: 1.32%; 95% CI: 0.81, 1.70) (Fig. 1a). Mortality declined over time with three distinct phases; it remained stable from 1988 to 1996 (APC: −0.47%; 95% CI: −0.13, 3.39), declined from 1996 to 2016 (APC: −0.55%; 95% CI: −0.78, −0.35), and decreased more sharply from 2016 to 2019 (APC: −3.42%; 95% CI: −6.96, −1.49) (Fig. 1b). Overall, incidence increased significantly (AAPC: 2.00%; 95% CI: 1.83, 2.17) and mortality declined (AAPC: −0.57%; 95% CI: −0.75, −0.37) (Supplementary Table S2).

**Table 4:** Annual percent change in renal cell carcinoma incidence and mortality rates, California Cancer Registry, 1988–2019.

	Incidence		Mortality	
	Period^a^	APC (95% CI)	Period^a^	APC (95% CI)
All				
	1988–1999	1.15 (−0.17, 1.82)	1988–1996	−0.47 (−0.13, 3.39)
	1999–2008	3.88 (3.19, 6.71)	1996–2016	−0.55 (−0.78, −0.35)
	2008–2019	1.32 (0.81, 1.70)	2016–2019	−3.42 (−6.96, −1.49)
Sex				
Male	1988–1999	0.94 (−0.82, 1.66)	1988–2016	−0.30 (−0.45, −0.06)
	1999–2008	3.65 (2.89, 7.00)	2016–2019	−4.55 (−10.03, −1.41)
	2008–2019	1.23 (0.63, 1.65)		
Female	1988–1998	1.04 (−2.35, 2.12)	1988–2019	−0.79 (−1.01, −0.56)
	1998–2008	3.89 (3.01, 8.16)		
	2008–2019	1.33 (0.21, 1.95)		
Age				
20–44 years	1988–1999	1.91 (−4.80, 4.02)	1988–2019	−0.62 (−1.27, 0.03)
	1999–2009	6.81 (5.24, 15.4)		
	2009–2019	3.30 (0.19, 4.57)		
45–54 years	1988–1998	−0.17 (−2.91, 1.21)	1988–2019	−2.12 (−2.54, −1.68)
	1998–2008	3.55 (2.62, 8.16)		
	2008–2019	1.74 (−0.27, 2.38)		
55–64 years	1988–2019	2.02 (1.84, 2.26)	1988–1996	0.49 (−1.38, 10.20)
			1996–2019	−1.78 (−6.15, −1.37)
65–74 years	1988–1998	1.01 (−2.24, 2.14)	1988–2016	−0.52 (−0.74, −0.14)
	1998–2008	4.01 (3.05, 8.36)	2016–2019	−6.54 (−14.38, −1.71)
	2008–2019	0.59 (−0.24, 1.23)		
75+ years	1988–1994	−1.87 (−8.07, 1.18)	1988–2007	0.94 (0.67, 1.41)
	1994–2009	3.33 (1.53, 5.96)	2007–2019	−0.35 (−1.16, 0.08)
	2009–2012	−3.04 (−5.23, 4.48)		
	2012–2019	2.36 (0.73, 6.32)		
Race and Ethnicity^b^				
Non-Hispanic White	1988–1999	1.21 (0.07, 1.82)	1988–2012	−0.48 (−0.64, 0.29)
	1999–2008	3.80 (3.09, 6.33)	2012–2019	−1.73 (−5.01, −0.79)
	2008–2019	0.71 (0.15, 1.14)		
Non-Hispanic Black	1988–2019	2.05 (1.66, 2.57)	1988–1996	3.49 (−0.29, 23.28)
			1996–2019	−1.28 (−8.88, −0.55)
Hispanic	1988–2001	1.76 (0.07, 3.52)	1988–1997	
	2001–2008	5.07 (−0.09, 8.68)	1997–2019	2.21 (0.24, 12.86)
	2008–2011	−0.71 (−2.27, 5.73)		−0.49 (−2.49, −0.10)
	2011–2019	2.67 (1.91, 4.61)		
Asian/Pacific Islander	1988–2004	1.52 (−5.13, 3.42)	1988–2015	1.10 (0.55, 2.23)
	2004–2007	10.56 (−0.86, 15.06)	2015–2019	−8.45 (−19.83, −2.31)
	2007–2019	0.83 (−1.36, 2.84)		
Stage				
Localized	1988–1998	2.31 (0.83, 3.31)		
	1998–2008	6.34 (5.60, 7.84)		
	2008–2019	1.51 (1.01, 1.97)		
Regional	1988–1999	−0.70 (−5.48, 0.53)		
	1999–2019	1.28 (0.89, 2.80)		
Distant	1988–2019	−0.10 (−0.31, 0.13)		
Unknown	1988–2019	−1.54 (−2.13, −0.93)		
nSES				
Quintile 1 (lowest)	2006–2019	2.62 (2.24, 3.05)		
Quintile 2	2006–2008	11.88 (2.31. 18.99)		
	2008–2019	0.8 (−1.83, 1.63)		
Quintile 3	2006–2017	2.14 (1.79, 3.34)		
	2017–2019	−2.72 (−5.63, 1.03)		
Quintile 4	2006–2019	1.38 (0.82, 1.97)		
Quintile 5	2006–2019	−0.10 (−0.65, 0.50)		

Abbreviations: APC, annual percent change; CI, confidence interval; nSES, neighborhood socioeconomic status.

a Time periods vary across subgroups because they reflect statistically significant trend changes identified by joinpoint regression. This method determines the best-fitting points where trends change in direction or magnitude, which may occur at different times for different subgroups.

b American Indian individuals, Alaska Native individuals, and individuals with unknown race/ethnicity were excluded due to insufficient sample size.

**Fig. 2:**
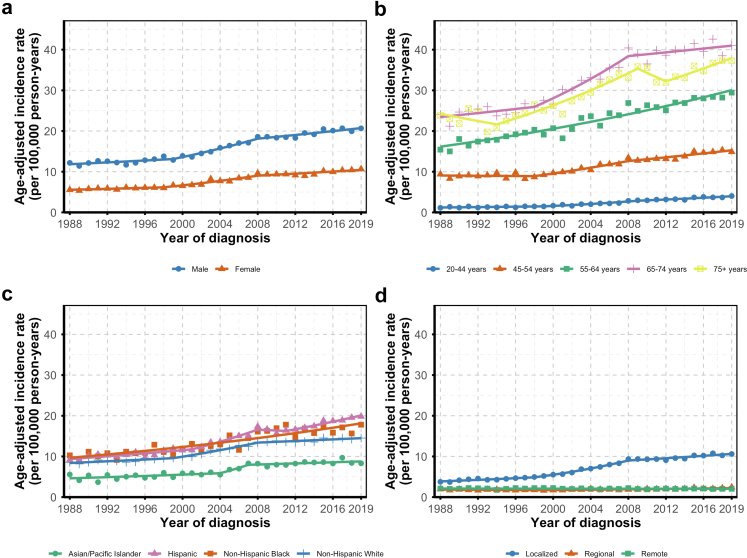
**Trends in renal cell carcinoma incidence rates stratified by sex (a), age at diagnosis (b), race and ethnicity (c), and stage at diagnosis (d), California Cancer Registry, 1988–2019**.

**Fig. 3:**
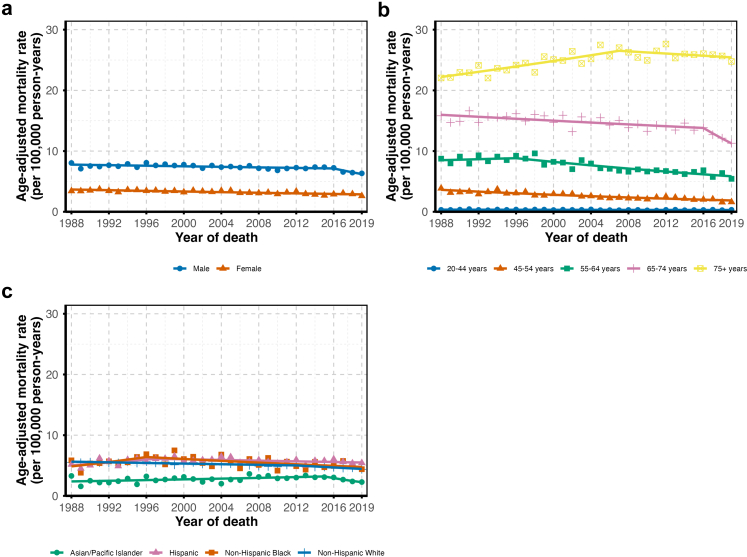
**Trends in renal cell carcinoma mortality rates stratified by sex (a), age at diagnosis (b), and race and ethnicity (c), California Cancer Registry, 1988–2019**.

Incidence rates were consistently higher in males but followed similar patterns in both sexes, with peak increases during the 1999–2008 period (Fig. 2a). Mortality declined steadily in males from 1988 to 2016 (APC: −0.30%; 95% CI: −0.45, −0.06) and more rapidly thereafter (APC: −4.55%; 95% CI: −10.03, −1.40). In females, mortality declined consistently throughout the period (APC: −0.79%; 95% CI: −1.01, −0.56 (Fig. 3a).

Incidence generally rose across all age groups, peaking around 2010, with slower increases thereafter (Fig. 2b). However, trends in the 75+ group were more variable, with alternating periods of decline and increase, though these were not statistically significant. Mortality declined across all age groups (Fig. 3b).

Stratification by race and ethnicity revealed varying RCC incidence and mortality trends (Figs. 2c and 3c). Non-Hispanic Black individuals experienced a steady incidence increase for the 1988–2019 period (APC: 2.05%; 95% CI: 1.66, 2.57), with mortality rising until the mid-1990s before then declining. Hispanic individuals showed a marked incidence increase from 2001 to 2008 (APC: 5.07%; 95% CI: −0.09, 8.68), albeit with a wide confidence interval, surpassing rates in non-Hispanic Black individuals. After a fairly stable period from 2008 to 2011, incidence rose again from 2011 to 2019 (APC: 2.67%; 95% CI: 1.91, 4.61). Mortality in Hispanic individuals increased until 2011 and declined thereafter. Non-Hispanic White individuals had consistent incidence growth, peaking between 1999 and 2008 (APC: 3.80%; 95% CI: 3.09, 6.33), with mortality declining steadily starting around 2012. While the incidence gap began to widen around 2008 for non-Hispanic Black and Hispanic individuals compared to non-Hispanic White individuals, the mortality gap narrowed over time among Black individuals and appeared to widen for Hispanic individuals. Asian and Pacific Islander individuals had a sharp but uncertain rise in incidence between 2004 and 2007 (APC: 10.56%; 95% CI: −0.86, 15.06), followed by an indication of stability through 2019 (APC: 0.83%; 95% CI: −1.36, 2.84). Overall, their incidence trends paralleled those of non-Hispanic White individuals but remained consistently lower. Their mortality increased until 2014, after which it declined; the mortality gap relative to other racial or ethnic groups widened over time.

Localized RCC incidence rose steadily, with an APC of 2.31% (95% CI: 0.83, 3.31) through 1998, which accelerated to 6.34% (95% CI: 5.60, 7.84) through 2008, before slowing to 1.51% (95% CI: 1.01, 1.97) through 2019 (Fig. 2d). Regional RCC incidence was stable or declined early on, followed by increases after 1999 (APC: 1. .28%; 95% CI: 0.89, 2.80). RCC diagnosed at the distant stage demonstrated no net change over the whole period (APC: −0.10%; 95% CI: −0.31, 0.13). The proportion of cases undergoing total and radical nephrectomies declined sharply starting in the early 2000s and eventually converged with the steadily increasing proportion of partial nephrectomies, which became more common over time (Supplementary Figure S3).

A gradient was observed in RCC incidence trends across nSES quintiles (Supplementary Figure S4). From 2006 to 2019, incidence rose most in the lowest SES group (quintile 1; APC: 2.62%; 95% CI: 2.24, 3.05). In contrast, incidence in the highest SES group (quintile 5) was the lowest and suggestive of stability (APC: −0.10%; 95% CI: −0.65, 0.50). The gap between the lowest and highest nSES groups steadily widened over time.

## Discussion

In this study, we used a California statewide registry to examine the incidence and mortality burden of RCC across age, sex, race and ethnicity, and nSES, and to update temporal trends (1988–2019) within groups defined by these sociodemographic factors. RCC incidence and mortality rates were highest among males, older age groups, American Indian or Alaska Native individuals, and those residing in lower SES neighborhoods. Overall, RCC incidence rates increased from 1988 through 2019, with faster increases and higher rates observed in Hispanic and non-Hispanic Black populations, as well as in individuals living in socioeconomically disadvantaged areas. Mortality trends revealed slower declines in RCC mortality rates among Hispanic and non-Hispanic Black individuals. Our results overall reflect persistent inequities in RCC detection and outcomes.

Understanding tumor characteristics and treatment trends is essential for evaluating progress in the RCC burden in California's diverse population. From 1988 through 2019, clear cell carcinoma was the most common histologic type. Additionally, 39.1% of cases lacked histologic specification, which reflects documented limitations in tumor characterization and reporting.^17^^,^^18^ Radical nephrectomy was the predominant primary treatment during the study period overall; partial nephrectomy—despite its nephron-sparing benefits for small, localized tumors^19^—was performed in only 23.3% of cases. Encouragingly, surgical trends showed a shift; while the proportion of individuals receiving total nephrectomies declined after the early 2000s, the percentage of partial nephrectomies steadily increased, reaching parity with total nephrectomies by 2019. This shift likely reflects greater adherence to guidelines and increased awareness of the benefits of partial nephrectomy in preserving renal function and reducing long-term complications, particularly for individuals with localized disease.^19^^,^^20^ Further progress is needed to ensure broader uptake of evidence-based surgical practices in RCC care.

Previous research in California found that RCC incidence rose from 1988 until around 2007–2009, after which it appeared to stabilize through 2013.^7^ In contrast, our findings showed that the incidence rate in California has continued to rise in recent years, aligning with national and international trends.^3^^,^^21^ This rise has been primarily driven by an increase in localized disease, which accounted for nearly two-thirds of all cases. This may be partly explained by the increased use of imaging in the late 1990s and early 2000s, which led to more frequent incidental detection of small renal tumors.^22^ That imaging use began to plateau around 2010,^23^ coinciding with a deceleration, but not a reversal, in the rise of incidence rates indicates that other factors may also be contributing. The parallel increase in the prevalence of established RCC risk factors, such as hypertension and obesity, indicates that broader changes in population risk factors may also have contributed to the sustained rise in incidence.^24^

It has been reported that mortality from RCC in the US has decreased by 0.6% per year from 1999 to 2020,^4^ consistent with our AAPC estimate. Additionally, our findings revealed that RCC mortality rates rose from 1988 to 1996 in California, peaking at approximately 5.5 deaths per 100,000 p-y, and then decreased to roughly 4.3 deaths per 100,000 p-y in 2019, which is still above the national average of 3.5 deaths per 100,000 p-y.^2^ While the downward trend suggests improvements in treatment, particularly for metastatic disease, the persistently elevated rate in the California population compared to the national average warrants further investigation.

Our analysis revealed pronounced racial and ethnic disparities in RCC burden, consistent with previous reports where non-Hispanic Black, Hispanic, and Native American populations experienced higher incidence and mortality rates than non-Hispanic White individuals.^25^, ^26^, ^27^ While non-Hispanic White individuals accounted for the highest absolute number of cases and deaths in California, reflecting their larger population size, our recent trends showed a relative slowdown in incidence rates and a more rapid decline in mortality rates. In contrast, Hispanic and non-Hispanic Black populations experienced higher and faster-rising incidence rates, coupled with slower declines in mortality rates. American Indian and Alaska Native populations faced the highest incidence and mortality rates relative to non-Hispanic White individuals. These findings echo the 2023 American Cancer Society report that underscored persistent inequities in cancer outcomes nationwide, likely driven by uneven access to early detection, slower dissemination of treatment advances, and differing risk factor profiles across groups — including higher rates of obesity and hypertension, and, in some populations, smoking — particularly in non-Hispanic Black and American Indian or Alaska Native individuals.^28^^,^^29^

The finding of a neighborhood socioeconomic gradient in the incidence of RCC is concerning. Individuals residing in the lowest nSES quintile experienced the highest and most sustained increases in RCC incidence, while those in the highest nSES had comparatively low and slightly declining rates. These patterns may reflect structural inequities, as residents of low-SES neighborhoods often have a higher prevalence of RCC risk factors, such as smoking, obesity, and hypertension.^30^ They may face limited access to healthcare resources and services—such as hypertension screening and management programs—that address known risk factors for RCC.^31^ Individuals living in low-SES neighborhoods may also have poor access to healthy foods or safe places for physical activity, which can promote weight gain and obesity.^32^ These limitations may also compound the racial and ethnic disparities observed in RCC incidence, as people of color are more likely to reside in disadvantaged neighborhoods.^33^^,^^34^ Residents in these areas also experience higher levels of emotional stress, which can take a long-term toll on the body, affecting immune, hormonal, and metabolic functions in ways that may increase the risk of developing cancer.^34^ These findings highlight the need for a more comprehensive investigation into the social and environmental factors underlying RCC disparities across socioeconomic position.

California provides a unique setting for this study because it is the most populous state, has high racial and ethnic diversity, exhibits extreme income inequality that may influence early detection and treatment access, and maintains a longstanding, rigorously managed cancer registry. To our knowledge, this is the first California-based study to document contemporaneous widening RCC incidence trends between low- and high-SES neighborhoods, as well as increasing incidence disparities among Non-Hispanic Black and Hispanic individuals compared to White individuals. Some limitations warrant consideration. Although the small number of American Indian and Alaska Native individuals in our dataset limited our ability to assess temporal trends, California remains one of the few states where it is possible to examine RCC incidence and mortality in this population. Some stratified estimates of trends were also based on limited sample sizes and had wide confidence intervals, introducing uncertainty. We interpreted our findings cautiously, emphasizing general patterns in the population rather than overinterpreting statistical significance, which could misrepresent the actual burden or trends. Overdiagnosis may also contribute to some of the observed differences in RCC incidence, particularly in high-SES areas where greater access to imaging may lead to the detection of indolent tumors. This could result in artificially higher incidence rates in these populations, making the observed difference in disease burden between groups appears smaller than the true difference. Lastly, joinpoint regression may have missed subtle or complex trend changes, such as gradual shifts, small fluctuations, or non-linear patterns, that did not meet the threshold for statistical significance or did not fit a segmented linear model. We interpreted the trends as a useful surveillance tool and an approximation of the underlying rate curve, recognizing that some nuanced changes may not have been detected.

RCC remains a significant public health concern in the US, with our study providing timely evidence of persistent and disproportionate burdens across socioeconomic and racial and ethnic groups. These findings underscore the relevance of RCC as a health equity issue and highlight the need for targeted public health responses. There is an urgent need to understand contextual social and built environment factors to better inform interventions on mitigating disparities in RCC incidence and mortality across nSES levels. Integrating data on structural and social drivers of health, alongside individual-level factors such as health behaviors, will be critical for identifying the root causes of these disparities, allocating appropriate resources, and informing targeted policies to reduce the RCC burden. Importantly, ongoing and enhanced surveillance will be necessary to monitor trends, evaluate whether efforts to reduce incidence align with projected declines,^3^ and determine if gains in survival are sustained.

## Contributors

KL contributed to the writing of the original draft, supervision, visualization, and writing, reviewing, and editing of the manuscript. KLin contributed to conceptualization, data curation, formal analysis, software, and writing, reviewing, and editing of the manuscript. DL, ZD, JTL, DYO, LCS, SLW, MVM, and JMC contributed to reviewing and editing. SJCS and MEL contributed to writing the original draft and to reviewing and editing. SG and IC contributed to conceptualization, reviewing, and editing. BIC and REG contributed to conceptualization, supervision, and reviewing and editing; REG also contributed to writing the original draft. KLin and REG accessed and verified the underlying data. All authors had full access to all the data in the study and had final responsibility for the decision to submit for publication.

## Data sharing statement

Access to CCR data is subject to approval and restrictions to protect patient confidentiality. Researchers may request access to these data through the CCR's data request process (https://www.ccrcal.org/retrieve-data/). The authors do not have the authority to share the data directly.

## Declaration of interests

SG has received research support from AstraZeneca; travel and accommodations from ASCO, Conquer Cancer Foundation, Horizon CME, and Meetings, Events and Conferences Coordinators Inc; and has consulted or advised for Puma Biotechnology, EMD Serono, Aveo, and Eisei on subject matter unrelated to this study. DYO has received research support from Merck, PACT Pharma, the Parker Institute for Cancer Immunotherapy, Poseida Therapeutics, TCR2 Therapeutics, Roche/Genentech, Nutcracker Therapeutics, Clasp Therapeutics, Janux Therapeutics, Amgen, and Allogene Therapeutics; travel and accommodations from Roche/Genentech, Poseida Therapeutics, and DAVA Oncology; and has consulted for Revelation Partners on subject matter unrelated to this study. LCS has received support for research from AstraZeneca and for advisory work from 23 & Me/Troper Wojcicki Foundation, both unrelated to this study. JTL has received grants and contracts from the U.S. Department of Veterans Affairs, the National Cancer Institute, and the U.S. Department of Defense for research unrelated to this project, as well as payment for expert testimony from the U.S. Department of Justice. MEL has received support paid to the institution from the Stanford Cancer Institute, the National Cancer Institute, and the National Institute of Diabetes and Digestive and Kidney Diseases for research unrelated to this project. REG consults for Hunton Andrews Kurth LLC on subject matter unrelated to this study. The authors otherwise declare no potential conflicts of interest.
