# Supplementary material for: Renal cell carcinoma incidence and mortality in California: a population-based study of sociodemographic patterns and temporal trends from 1988 through 2019
Source: Lancet Reg Health Am. 2026 Feb 27;56:101430. doi: 10.1016/j.lana.2026.101430 (PMC12964298; doi:10.1016/j.lana.2026.101430)

Supplementary Materials

Title: Renal cell carcinoma incidence and mortality in California: A population-based study of sociodemographic patterns and temporal trends from 1988 through 2019

Authors: Kevin L'Espérance, Katherine Lin, Daphne Lichtensztajn, Simon John Christoph Soerensen, Shuchi Gulati, Zhengyi Deng, John T. Leppert, David Y. Oh, Lori C. Sakoda, Samuel L. Washington III, Maxwell V. Meng, June M. Chan, Marvin E. Langston, Iona Cheng, Benjamin I. Chung, Rebecca E. Graff

Section 1. Incidence rate ratios by population demographics *2*

Supplementary Figure 1. Forest plot of incidence rate ratios by population demographics, California Cancer Registry, 1988-2019. ..... **2**

Section 2. Neighborhood socioeconomic status gradient by renal cell carcinoma stage *3*

Supplementary Table 1. Age-adjusted incidence rate ratios of primary renal cell carcinoma across neighborhood socioeconomic status by stage at diagnosis, California Cancer Registry, 2006-2019 ..... **3**

Section 3. Mortality rate ratios by population demographics *4*

Supplementary Figure 2. Forest plot of mortality rate ratios by population demographics, California Cancer Registry, 1988-2019. ..... **4**

Section 4. Average annual percent change *5*

Supplementary Table 2. Average annual percent change in renal cell carcinoma incidence and **5**

mortality rates, California Cancer Registry, 1988–2019 ..... **5**

Section 5. Temporal change in the proportion of primary treatment types in individuals with renal cell carcinoma *6*

Supplementary Table 3. Annual percent change in primary treatment types among individuals with renal cell carcinoma, California Cancer Registry, 1988-2019 ..... **6**

Supplementary Figure 3. Trends in the proportions of primary treatment types among individuals with renal cell carcinoma, California Cancer Registry, 1988-2019 ..... **7**

Section 6. Temporal change in incidence by neighborhood socioeconomic status *8*

Supplementary Figure 4. Trends in renal cell carcinoma incidence rates stratified by neighborhood socioeconomic status, California Cancer Registry, 1988-2019 ..... **8**

Section 1. Incidence rate ratios by population demographics

Supplementary Figure 1. Forest plot of incidence rate ratios by population demographics, California Cancer Registry, 1988-2019.

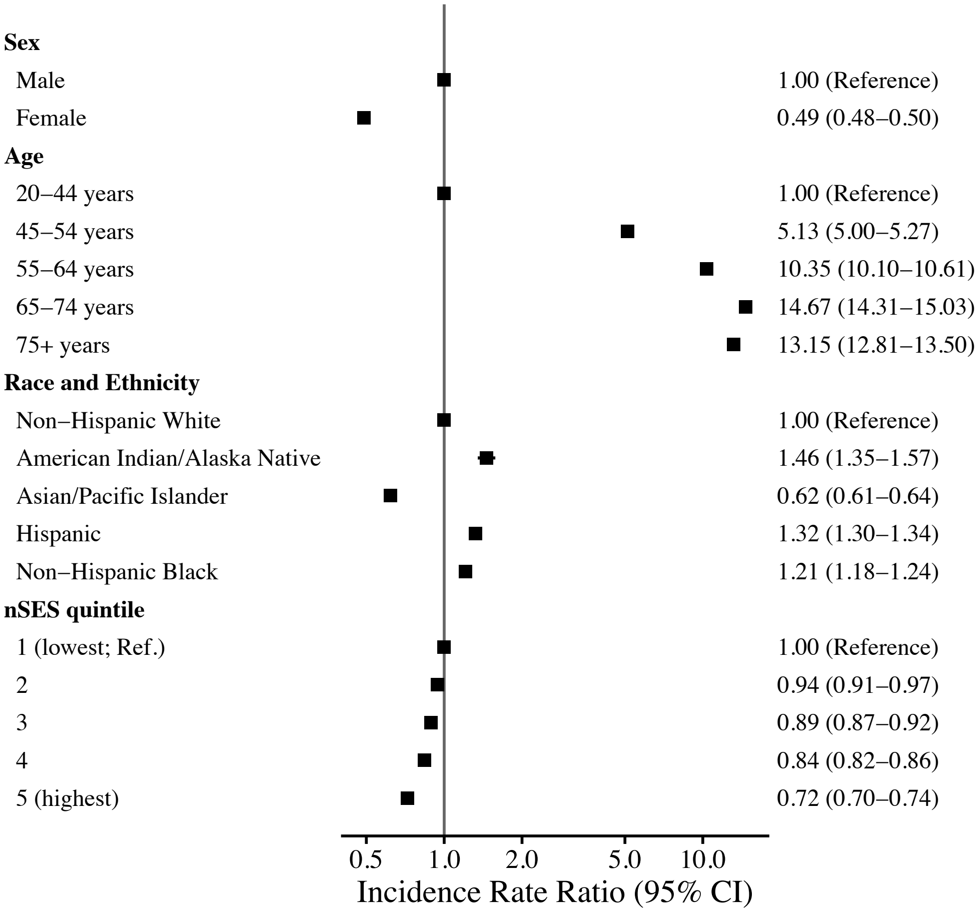

Section 2. Neighborhood socioeconomic status gradient by renal cell carcinoma stage

Supplementary Table 1. Age-adjusted incidence rate ratios of primary renal cell carcinoma across neighborhood socioeconomic status by stage at diagnosis, California Cancer Registry, 2006-2019

	Stage at diagnosis			
	**Localized**	**Regional**	**Remote**	**Unknown**
nSES quintile	IRR (95% CI)	IRR (95% CI)	IRR (95% CI)	IRR (95% CI)
1 (lowest)	1.00 (ref.)	1.00 (ref.)	1.00 (ref.)	1.00 (ref.)
2	0.96 (0.93-1.00)	0.92 (0.86-0.99)	0.91 (0.85-0.97)	0.75 (0.64-0.88)
3	0.92 (0.89-0.96)	0.91 (0.85-0.98)	0.81 (0.76-0.87)	0.65 (0.56-0.76)
4	0.88 (0.85-0.91)	0.87 (0.81-0.93)	0.72 (0.67-0.77)	0.56 (0.48-0.66)
5 (highest)	0.78 (0.75-0.81)	0.70 (0.65-0.75)	0.58 (0.53-0.62)	0.40 (0.34-0.48)
p-trend	0.010	0.028	0.002	0.002

Abbreviations: CI, confidence interval; IRR, incidence rate ratio; nSES, neighborhood socioeconomic status

Section 3. Mortality rate ratios by population demographics

Supplementary Figure 2. Forest plot of mortality rate ratios by population demographics, California Cancer Registry, 1988-2019.

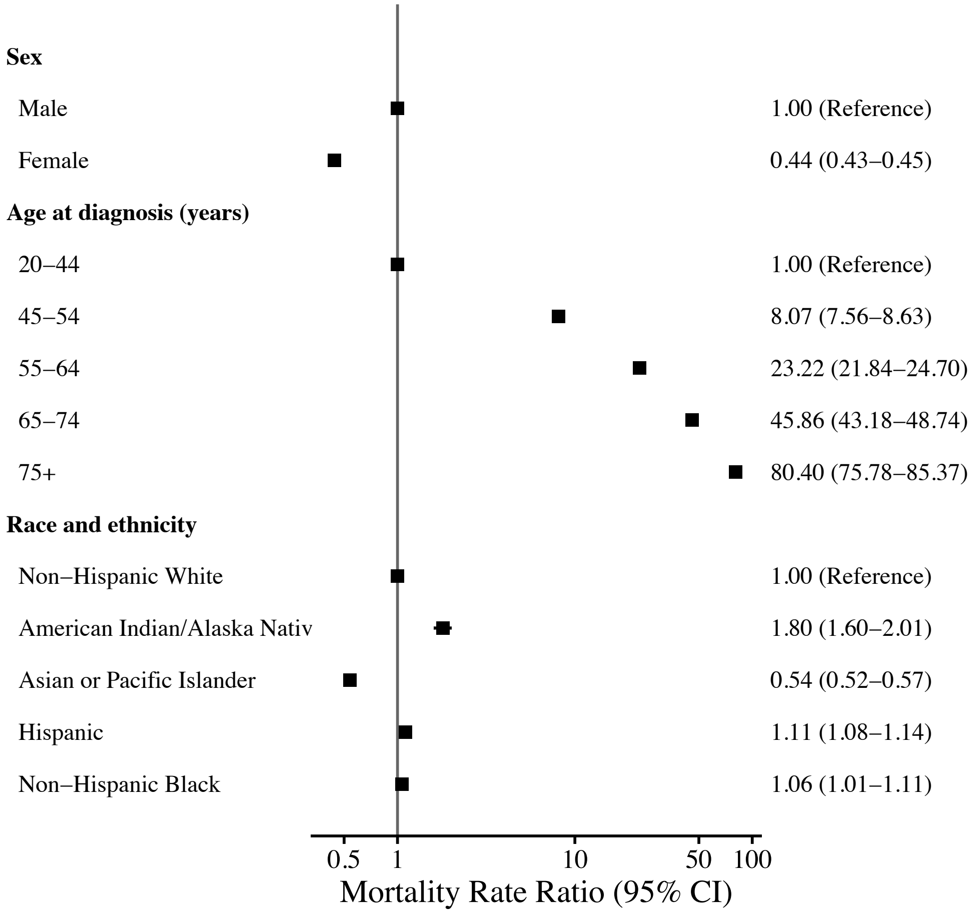

Section 4. Average annual percent change

Supplementary Table 2. Average annual percent change in renal cell carcinoma incidence and mortality rates, California Cancer Registry, 1988–2019

	Incidence	Mortality
	AAPC (95% CI)	AAPC (95% CI)
All	2.00 (1.83; 2.17)	-0.57 (-0.75; -0.37)
Sex		
Male	1.82 (1.63; 2.01)	-0.72 (-1.00; -0.49)
Female	2.05 (1.76; 2.32)	-0.79 (-1.01; -0.56)
Age		
20-44	3.92 (3.34; 4.49)	-0.62 (-1.27; 0.03)
45-54	1.70 (1.38; 1.99)	-2.12 (-2.54; -1.68)
55-64	2.02 (1.84; 2.26)	-1.20 (-1.84; -0.56)
65-74	1.82 (1.55; 2.09)	-1.12 (-1.55; -0.77)
75+	1.46 (1.10; 1.86)	0.44 (0.28; 0.62)
Race and ethnicity		
Non-Hispanic White	1.78 (1.61; 1.94)	-0.76 (-0.98; -0.54)
Non-Hispanic Black	2.05 (1.66; 2.57)	-0.07 (-1.23; 1.15)
Hispanic	2.49 (2.27; 2.77)	0.29 (-0.24; 1.00)
Asian or Pacific Islander	2.09 (1.46; 2.87)	-0.18 (-0.84; 0.69)
Quintiles of nSES [c]		
1 (lowest)	2.62 (2.24; 3.05)	
2	2.45 (1.21; 3.37)	
3	1.38 (0.94; 1.98)	
4	1.38 (0.82; 1.97)	
5 (highest)	-0.10 (-0.65; 0.50)	
Stage		
Localized	3.31 (3.10; 3.53)	
Regional	0.58 (0.20; 0.96)	
Remote	-0.10 (-0.31; 0.13)	
Unknown	-1.54 (-2.13; -0.92)	
Primary treatment		
Excision/Partial Nephrectomy	12.43 (11.66; 13.34)	
Total/Radical Nephrectomy	0.48 (0.27; 0.72)	
Surgery not otherwise specified	-3.67 (-4.20; -3.16)	
No surgery	1.20 (1.06; 1.41)	

Abbreviations: AAPC, average annual percent change; CI, confidence interval; nSES, neighborhood socioeconomic status

Section 5. Temporal change in the proportion of primary treatment types in individuals with renal cell carcinoma

Supplementary Table 3. Annual percent change in primary treatment types among individuals with renal cell carcinoma, California Cancer Registry, 1988-2019

	Proportion	
Primary Treatment	**Period [a]**	**APC (95% CI)**
Excision/Partial Nephrectomy	1988–1996	5.55 (-3.66; 9.91)
	1996–2000	29.62 (20.53; 41.54)
	2000–2011	12.32 (11.46; 13.11)
	2011–2019	2.27 (1.40; 2.84)
Total/Radical Nephrectomy	1988–2002	-0.20 (-0.40; 0.11)
	2002–2008	-2.00 (-2.73; -1.21)
	2008–2011	-6.87 (-7.77; -4.94)
	2011–2019	-2.09 (-2.58; -1.18)
Surgery Not Otherwise Specified	1988–2019	-5.86 (-6.39; -5.37)
No surgery	1988–1999	0.30 (-1.73; 1.55)
	1999–2002	-8.27 (-9.92; 2.30)
	2002–2008	-1.50 (-7.45; 0.88)
	2008–2019	0.98 (0.26; 2.89)

Abbreviations: APC, annual percent change
[a] Time periods vary across subgroups because they reflect statistically significant trend changes identified by joinpoint regression. This method determines the best-fitting points where trends change in direction or magnitude, which may occur at different times for different subgroups.

Supplementary Figure 3. Trends in the proportions of primary treatment types among individuals with renal cell carcinoma, California Cancer Registry, 1988-2019

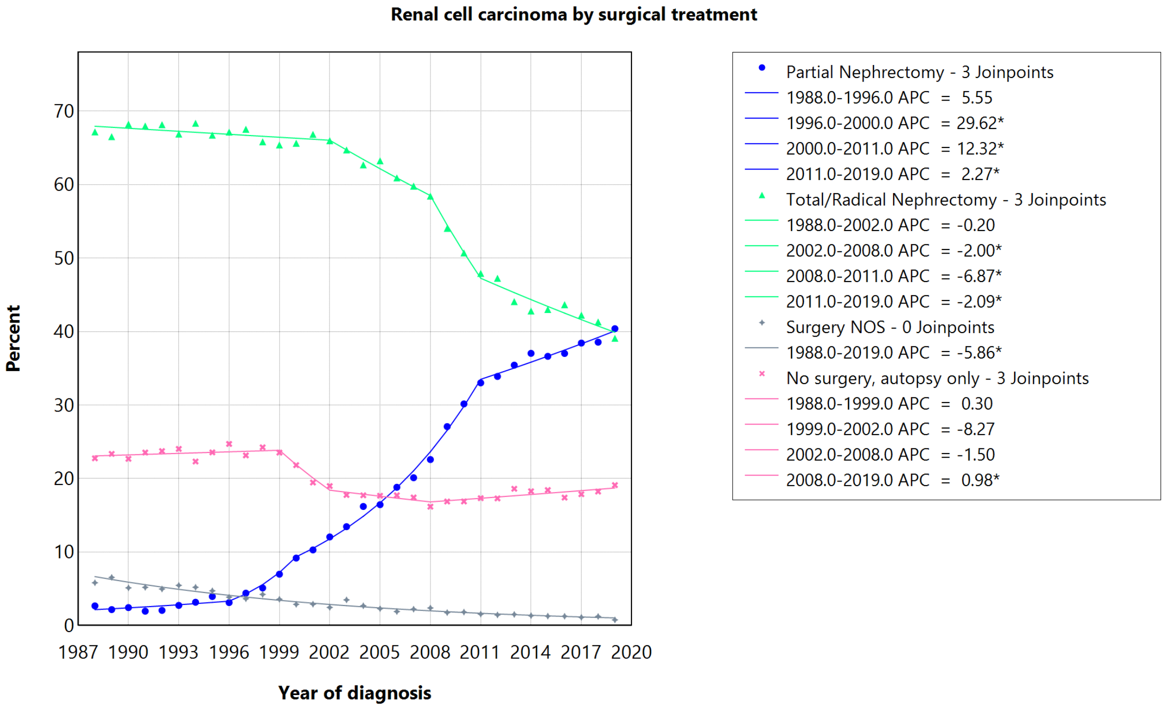

Section 6. Temporal change in incidence by neighborhood socioeconomic status

Supplementary Figure 4. Trends in renal cell carcinoma incidence rates stratified by neighborhood socioeconomic status, California Cancer Registry, 1988-2019

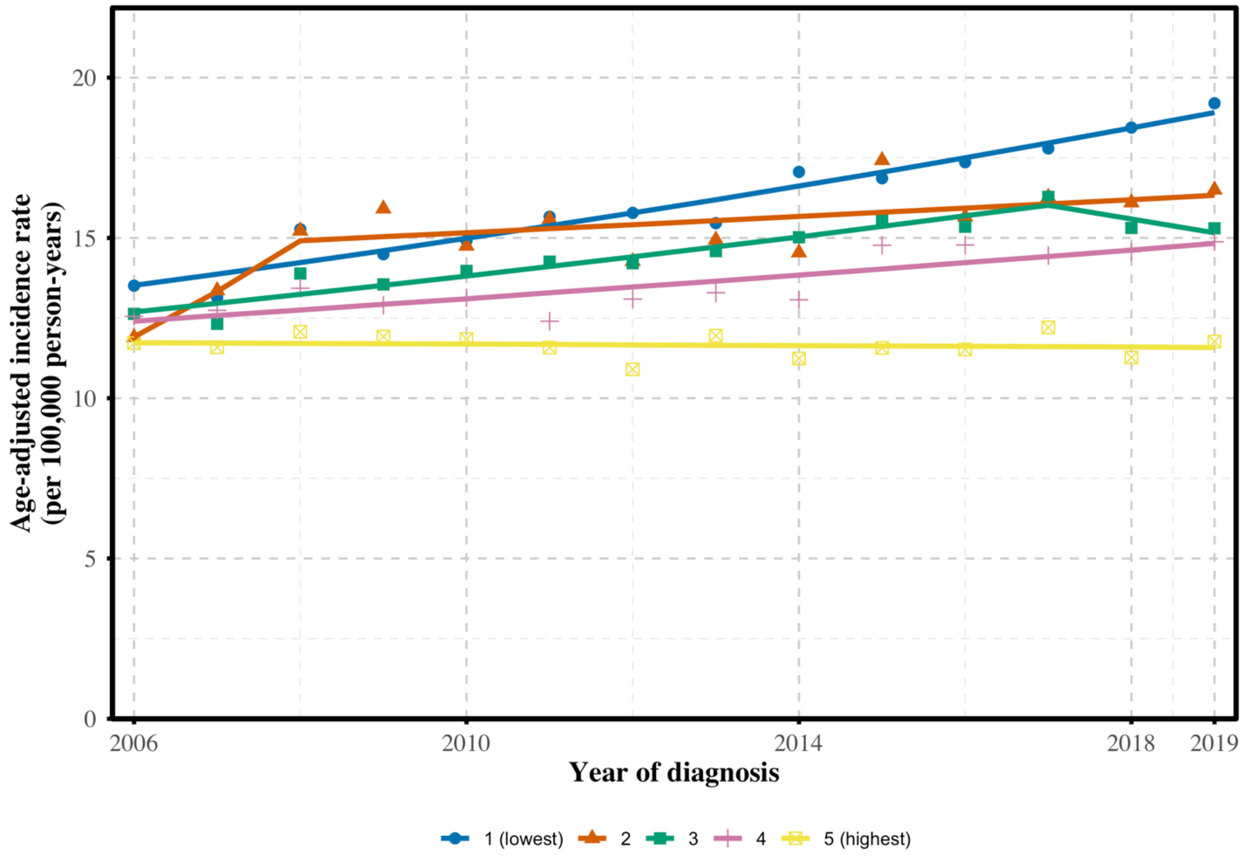

Supplement: Supplementary Materials [file mmc1.pdf]
